# Sulfur dioxide-induced guard cell death and stomatal closure are attenuated in nitrate/proton antiporter *AtCLCa* mutants

**DOI:** 10.1093/pcp/pcaf042

**Published:** 2025-06-12

**Authors:** Lia Ooi, Takakazu Matsuura, Izumi C Mori

**Affiliations:** Institute of Plant Science and Resources, Okayama University, 2-20-1 Chuo, Kurashiki, Okayama 710-0046, Japan; Plant & Microbial Research Unit, Research, Technology & Value Creation Division, Nagase Viita Co., Ltd., 675-1 Fujisaki, Naka-ku, Okayama 702-8006, Japan; Institute of Plant Science and Resources, Okayama University, 2-20-1 Chuo, Kurashiki, Okayama 710-0046, Japan; Institute of Plant Science and Resources, Okayama University, 2-20-1 Chuo, Kurashiki, Okayama 710-0046, Japan

**Keywords:** airborne pollutants, cytosolic acidification, stomatal closure, sulfur dioxide

## Abstract

Guard cells surrounding the stomata play a crucial role in regulating the entrance of hazardous gases such as SO_2_ into leaves. Stomatal closure could be a plant response to mitigate SO_2_ damage, although the mechanism for SO_2_-induced closure remains controversial. Proposed mediators for SO_2_-induced stomatal closure include phytohormones, reactive oxygen species, gasotransmitters, and cytosolic acidification. In this study, we investigated the mechanism of stomatal closure in *Arabidopsis* in response to SO_2_. Despite an increment in auxin and jasmonates after SO_2_ exposure, the addition of auxin did not cause stomatal closure and jasmonate-insensitive mutants exhibited SO_2_-induced stomatal closure suggesting auxin and jasmonates are not mediators leading to the closure. In addition, supplementation of scavenging reagents for reactive oxygen species and gasotransmitters did not inhibit SO_2_-induced closure. Instead, we found that cytosolic acidification is a credible mechanism for SO_2_-induced stomatal closure in *Arabidopsis*. *CLCa* mutants coding H^+^/nitrate antiporter, involved in cytosolic pH homeostasis, showed less sensitive stomatal phenotype against SO_2_. These results suggest that cytosolic pH homeostasis plays a tenable role in SO_2_ response in guard cells.

## Introduction

Stomatal pores facilitate gas exchange within the epidermis in vascular plants. These pores are composed of a pair of guard cells. The turgor regulation of guard cells drives stomatal movements in response to environmental changes (Hetherrington and Woodward 2003). Stomata play a crucial role not only in the absorption of carbon dioxide for photosynthesis and the dissipation of water from leaves but also in serving as entry points for air pollutants. Ozone, nitrogen dioxide, and sulfur dioxide (SO_2_) can invade leaves through stomatal pores, causing damage to foliage at elevated levels.

Stomatal closure plays a role in defense to air pollutants. An earlier study demonstrated that induction of artificial stomatal closure made plants resistant to SO_2_ fumigation (Kondo and Sugahara 1978). In the same study, it was reported that there was a significant variability in SO_2_ sensitivity among plant species, which showed a positive correlation with abscisic acid (ABA) content in the leaves. In a field study conducted in a heavily air-polluted area, it was observed that higher levels of SO_2_ pollution were associated with reduced stomatal conductance in tropical trees (Rao and Dubey 1990). These findings suggest that stomatal closure is crucial for mitigating the impact of hazardous gases in plants (Majernik and Mansfield 1970, Taylor 1978).

It has been noted that the response of stomata to SO_2_ is complex. Leaf age, stomatal position, and sulfur availability within leaves can lead to different response of stomata to SO_2_, even within the same plant (Olszyk and Tibbitts 1981a, 1981b, Mozhgani et al. 2024). A study conducted on *Helianthus annuus* (common sunflower) demonstrated that when SO_2_ damage is mild, it can be reversed. For instance, stomata which close upon SO_2_ exposure can reopen after a few hours post-exposure. However, when the damage is severe, it became irreversible (Omasa et al. 1985). This suggests a correlation between the extent of damage and the degree of stomatal opening. Irreversible leaf injury is characterized by cell collapse and leaf wilting. The mechanisms responsible for these effects in response to SO_2_ exposure remained to be revealed. Furthermore, the signaling mechanisms involved in SO_2_ response in guard cells remained poorly understood.

Several reports have highlighted the potential role of ABA in regulating the process of SO_2_-induced stomatal closure. External application of ABA has been shown to induce stomatal closure, resulting in increased resistance to SO_2_ (Kondo and Sugahara 1978). Additionally, ABA levels in *Glycine max* (soybean) leaves were found to increase in response to SO_2_ fumigation in concentration- and time-dependent manners (Gupta et al. 1991). An analysis of SO_2_ resistance in *Coleus scutellarioides* cultivars revealed a correlation between higher ABA levels, reduced transpiration rates, and fewer SO_2_-induced lesions (Krizek et al. 2001). While the involvement of numerous phytohormones in regulating stomatal aperture has gained recognition (Murata et al. 2015), the potential roles of plant hormones in SO_2_-induced stomatal closure have not been thoroughly explored, apart from ABA.

Lipid and protein oxidation are also proposed as mechanisms for SO_2_ toxicity in plants. A study reported that the occurrence of hydrogen peroxide and singlet oxygen molecules in strawberry leaves following SO_2_ exposure leads to plant damages (Muneer et al. 2014). Numerous reports have demonstrated that superoxide detoxification by superoxide dismutase is a critical step in enhancing SO_2_ resistance in various plant species (Shimazaki et al. 1980, Tanaka and Sugahara 1980, Rao and Dubey 1990, Madamanchi and Alscher 1991, Madamanchi et al. 1994). A deficiency in ascorbic acid has also been linked to increased sensitivity to SO_2_ (Conklin et al. 1996). It can be hypothesized that oxidative damage may also serve as a potential trigger for stomatal closure, similar as observed in ozone exposure (Vainonen and Kangasjarvi 2015).

Hu et al. (2014) reported that nitric oxide (NO) scavenger, 2-(4-carboxyphenyl)-4,4,5,5-tetramethylimidazoline-1-oxyl-3-oxide (cPTIO), and hydrogen sulfide (H_2_S) scavenger, hypotaurine, were able to block SO_2_-induced stomatal closure in *Ipomoea batatas*. This study implicates that SO_2_ signaling mechanism in guard cells is mediated by two gasotransmitters, NO and H_2_S. In *Hemerocallis fulva*, mitigation of SO_2_-induced guard cell death by treatments with catalase and ascorbate, suggesting the role of ROS in this process (Wei et al. 2013). This study also reported the NO scavenger, cPTIO and Ca^2+^ channel blocker, LaCl_3_, decreased cell death rate. The generality of the involvement of these gasotransmitters in SO_2_-induced stomatal closure in other plant species remains to be evaluated.

Open Stomata 1 (OST1) is a SnRK2-type serine/threonine kinase. It plays a critical role in induction of stomatal closure in response to myriad environmental and endogenous stimuli, such as methyl jasmonate (MeJA), microbe-associated molecular pattern, and ozone (Vahisalu et al. 2010, Ye et al. 2015, Yin et al. 2016), as well as ABA (Mustilli et al. 2002). However, our previous study (Ooi et al. 2019) demonstrated that the loss-of-function mutations in *OST1* did not affect SO_2_ response of stomata. This suggests that the mechanism for SO_2_-induced stomatal closure is distinct from that for those stimuli mediated with OST1 kinase.

NADPH oxidases play critical roles in reactive oxygen species (ROS) signaling in plants in addition to the roles in ROS burst during defense response (Fluhr 2009). Respiratory burst oxidase homologs, RbohD and RbohF are reported to participate in ABA- and methyl jasmonate-induced stomatal closure (Kwak et al. 2003, Suhita et al. 2004). Our previous report showed that SO_2_-induced stomatal closure was not impaired in the *rbohd rbohf* double mutant of Arabidopsis (Ooi et al. 2019). This result is one of the negative pieces of evidence for the involvement of oxidative signals in SO_2_-induced stomatal closure.

Cell collapse is a characteristic of SO_2_ damage in plant cells. In our previous study, we showed that cell death of guard cells is likely associated with stomatal closure (Ooi et al. 2019). This cell death is not related to chromosome fragmentation, suggesting it may be a form of necrotic cell death rather than apoptotic cell death. That study postulated that the mechanism behind the collapse of guard cells induced by SO_2_ involves cytosolic acidification, although further assessment is needed to confirm this hypothesis. *CLORIDE CHANNEL A* (*CLCa*) gene in *Arabidopsis* codes a proton-nitrate antiport protein localized in vacuolar membrane (De Angeli et al. 2006). This antiporter is not only involved in nitrate accumulation, but also involved in pH homeostasis of guard cells (Demes et al. 2020). It is a good candidate to confirm our hypothesis.

Here, we investigated the involvements of phytohormones, ROS, gasotransmitters, and cytosolic acidification in SO_2_-induced stomatal closure. To examine the involvement of plant hormones in SO_2_-induced stomatal closure, we took advantage of simultaneous multiple hormone analysis using liquid chromatography–mass spectrometry (LC–MS) in SO_2_-exposed *Arabidopsis* leaves. Genetic mutants related to the hormones demonstrated changes in the contents were analyzed to address the involvement of phytohormones in the process. The involvement of productions of ROS in SO_2_-induced stomatal closure was assessed using two ROS scavengers, *N*-acetylcysteine (NAC) and 1,2-dihydroxybenzene-3,5- disulfonic acid (tiron). We also examined the effects of the NO scavenger, cPTIO, and the H_2_S scavenger, hypotaurine to investigate the contribution of NO and H_2_S in SO_2_-induced stomatal closure in *Arabidopsis*. Furthermore, taking advantage of the cytosolic pH homeostasis-impaired phenotype of *clca* mutants, we tested the hypothesis of cytosolic acidification as a critical mechanism responsible for guard cell death induced by SO_2_ exposure.

## Materials and Methods

### Plant materials and growth conditions

*Arabidopsis thaliana* wild types (ecotypes Col and WS), *coronatine insensitive 1* (*coi1*) mutant (Munemasa et al. 2007), and *chloride channel-a* mutants, *clca2* and *clca3* (Wege et al. 2014, Mozhgani et al. 2024) were grown in pots filled with Vermiculite GS (Nittai Co. Ltd., Osaka) and seedling soil (SK Agri, Kiryu, Japan) in a 4:3 ratio, in a growth chamber (Biotron LPH 200, NK System, Osaka) with 16 h-light/8 h-dark photoperiod regime at 135 µmol/m^2^/s^–1^, 23 ± 0.5°C, and 65–80% relative humidity.

### Chemicals

All chemicals used in this study were of the special grade or the highest quality grade procured from Nacalai Testque, Inc. (Kyoto, Japan), Fujifilm Wako Pure Chemical Corporation (Osaka, Japan), or Kanto Chemical Co., Inc. (Tokyo, Japan), unless stated otherwise.

### Phytohormone quantification

The contents of nine phytohormones, GA_1_, GA_4_, SA, JA, JA-Ile, IAA, tZ, iP, and ABA, in excised leaves treated with 180 min aqueous SO_2_ solution were quantified by LC–MS as previously described (Cho et al. 2022).

### Stomatal closure assay

Measurement of stomatal aperture width was conducted essentially as previously described (Ooi et al. 2019). In brief, excised rosette leaves of 4- to 6 week-old plants were pre-incubated for 2 h floated on stomatal opening solution containing 5 mM KCl, 50 µM CaCl_2_, and 10 mM 2-(*N*-morpholino)ethanesulfonic acid-tris(hydroxymethyl)aminomethane (pH 5.7) under white fluorescent tube illumination at 120 µmol/m^2^/s, followed by a 3-h treatment of aqueous SO_2_ added in the stomatal opening solution, unless stated otherwise. Concentrations of H_2_SO_3_ in the solution were estimated as previously described (Ooi et al. 2019). After H_2_SO_3_ treatment, epidermal fragments were released from SO_2_-treated leaves by blending using a Waring Bar Blender (BB-900, Waring Products Inc., Torrington, CT, USA). Stomatal aperture width was measured under a microscope (BA300, Shimadzu Rika Corporation, Kyoto, Japan). Concentrations of the protonated forms of formic acid and maleic acid were estimated from their p*K*_a1_ values and pH of buffering solution (Supplemental Table S1).

### Treatments with H_2_S, NO, and ROS scavengers

Excised rosette leaves were pre-incubated for 2 h, under white fluorescent tube illumination at 120 µmol/m^2^/s, in stomata opening solution supplemented with the H_2_S scavenger, hypotaurine (Merck KGaA, Darmstadt, Germany), the NO scavenger, cPTIO (Dojindo Laboratories, Kumamoto, Japan), the ROS scavengers, tiron (Tokyo Chemical Industry Co. Ltd., Tokyo, Japan) or NAC before they were exposed to H_2_SO_3_ for 2 h. Measurement of stomatal aperture width was conducted as described earlier.

### Guard cell viability test

The viability of guard cells was assessed as previously described (Ooi et al. 2019). In brief, released epidermal fragments from SO_2_-treated excised rosette leaves were double-stained with 50 ng/ml of carboxyfluorescein diacetate (CFDA, Molecular Probes, Eugene, OR, USA) for 20 min and 2 ng/µl of propidium iodide (PI, Life technologies, Eugene, OR, USA) for 10 min in the stomatal opening solution. Stained epidermal fragments were thoroughly rinsed with distilled water and observed under a fluorescence microscope (Biozero BZ-X700, Keyence Corporation, Osaka, Japan) with BZ-X filter GFP for CFDA (excitation wavelength: 470/40 nm, emission wavelength: 525/50 nm, dichroic mirror wavelength: 495 nm) and BZ-X filter TRITC for PI (excitation wavelength: 545/25 nm, emission wavelength: 605/70 nm, dichroic mirror wavelength: 565 nm, Keyence Corporation). CFDA-positive and PI-positive cells were counted as viable cells and dead cells, respectively.

## Results

### Potential involvement of phytohormones in SO_2_-induced stomatal closure

**Figure 1.**
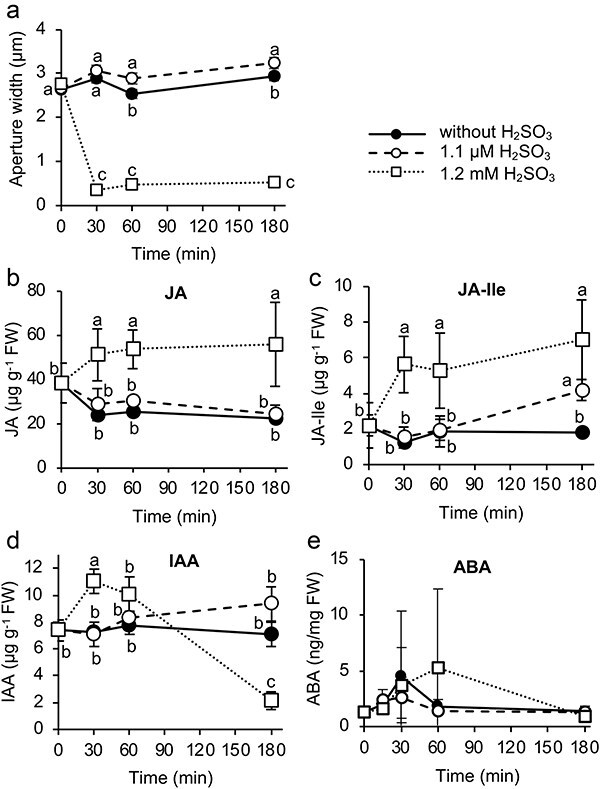
Stomatal closure induction and hormone contents in H_2_SO_3_-treated leaves. (a) Time course of stomatal aperture width in a period of 180 min incubation in H_2_SO_3_; Contents of (b) jasmonic acid (JA), (c) jasmonoyl-isoleucine (JA-Ile), (d) indoleacetic acid (IAA) and (e) abscisic acid (ABA) in H_2_SO_3_-treated leaves. Mature rosette leaves of wild-type plants were incubated in stomata opening buffer containing 0 (control), 1.1 μM, and 1.2 mM H_2_SO_3_ for 180 min under white light radiation. Error bars represent SD. Some error bars are too small to be seen. Lower case letters indicate significant differences at 0.05 via one-way ANOVA followed by Tukey’s honestly significant post hoc test at each exposure time.

In our previous report, we identified that when SO_2_ diffused into water, three chemical species H_2_SO_3_, HSO_3_^−^, and SO_3_^2−^ are formed; H_2_SO_3_ is the sole SO_2_-derived species which is responsible for SO_2_-induced stomatal closure (Ooi et al. 2019). An earlier study in various crop plants postulated that SO_2_-induced stomatal closure was mediated by ABA (Kondo and Sugahara 1978), while this statement requires further clarification. To investigate the roles of plant hormones in the process of SO_2_-induced stomatal closure, we quantified the contents of nine plant hormones in excised leaves exposed to H_2_SO_3_ for a period of 3 h, by LC–MS. We chose two H_2_SO_3_ concentrations (1.1 µM and 1.2 mM) for SO_2_ exposure here, since these concentrations resulted in distinct response in stomatal behavior: a slight widening (1.1 µM) and substantial closure of stomatal aperture (1.2 mM), respectively (Fig. 1A).

The contents of the major active gibberellins (gibberellinA_1_ [GA_1_], gibberellin A_4_ [GA_4_]), free salicylic acid (SA), and major active cytokinins (*trans*-zeatin [tZ], *N*^6^-isopentenyl adenine [iP]) in the leaves did not show significant changes by either 1.1 µM or 1.2 mM H_2_SO_3_ exposure (Supplementary Fig. S1). Therefore, the potential involvement of these five natural plant hormones in stomatal response to SO_2_ was inferred as less probable. Contents of jasmonates (jasmonic acid [JA], jasmonoyl-isoleucine [JA-Ile]) changed upon H_2_SO_3_ treatments (Fig. 1B and C). In the first 30 min, JA and JA-Ile contents decreased from the initial value in the control and 1.1 µM H_2_SO_3_ treatments. This may be attributed to the recovery from injury by leaves excision, which transiently increases JA and JA-Ile contents. For 1.2 mM H_2_SO_3_ treatment, JA and JA-Ile contents elevated significantly at 30 min and the elevation persisted for at least 180 min. At 1.1 µM H_2_SO_3_, the increase in JA contents was not observed, while JA-Ile contents increase slightly at 180 min. Auxin (indole-3-acetic acid [IAA]) contents were not significantly different at 1.1 µM H_2_SO_3_ compared to the control (Fig. 1D). Nevertheless, 1.2 mM-H_2_SO_3_ treatment caused a significant increase in IAA contents at 30 min and decreased to a lower level below the original level, at 180 min. The contents of ABA transiently increased after a 60 min treatment with 1.2 mM H_2_SO_3_, while it was not statistically significant (Fig. 1E).

**Figure 2.**
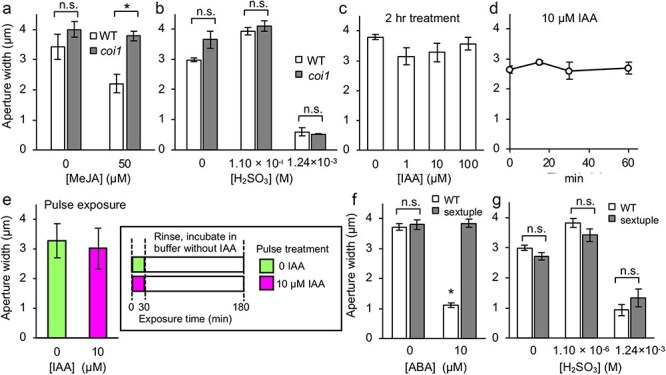
Induction of stomatal closure by meJA, IAA and ABA in wild type (Col), and MeJA- and ABA-insensitive mutants (*coi1* and *sextuple*). (a) MeJA-induced stomatal closure in Col and the jasmonate-insensitive *coronatine-insensitive 1* (*coi1*) mutant. Four biological replicates consisted of 80 stomata. (b) Stomatal closure induction by H_2_SO_3_ in Col and *coi1*. Four biological replicates consisted of 80 stomata. (c) Stomatal movement of Col against 2 h of 0–100 µM IAA incubation. 0 IAA indicates 2 h treatment with 0.1% ethanol as the solvent control; eight biological replicates consisted of 160 stomata. (d) Stomatal movement of Col against 0, 15, 30, and 60 min of 10 µM IAA incubation. Four biological replicates consisted of 80 stomata. (e) Stomatal closure induction by pulse treatment of 10 µM IAA. Pre-incubated leaves of Col were treated with 30 min of 10 µM IAA in stomata opening buffer, rinsed with distilled water, followed by 150 min of incubation without IAA before the measurement of stomatal aperture width; three biological replicates consisted of 60 stomata. Insert panel illustrates the overview of experimental procedure. (f) ABA-induced stomatal closure in Col and sextuple mutant of the ABA receptor genes (*sextuple*). Three biological replicates consisted of 60 stomata. (g) Stomatal closure induction by H_2_SO_3_ in Col and *sextuple*; three biological replicates consisted of 60 stomata. Error bars indicate standard error of the mean. Dunnett’s tests (α = 0.05) performed on (c) and (d), and Student’s *t*-test (*P* > .05) conducted for data in (b) and (e) found no significant differences as compared to the respective controls.

In accordance with our hormone quantification results, we explored the potential participation of jasmonates and ABA in SO_2_-induced stomatal closure utilizing hormone-insensitive mutants. We also examined the involvement of IAA in stomatal closure induction utilizing exogenous application of IAA (Fig. 2). H_2_SO_3_ treatment of the JA-insensitive mutant, *coronatine-insensitive 1* (*coi1*), showed no significant difference from the wild type. Reportedly, stomata of *coi1* did not close by the application of meJA (Fig. 2A). The lower concentration (1.1 µM) of H_2_SO_3_ slightly increased the stomatal aperture width and the higher concentration (1.2 mM) induced substantial stomatal closure both in the wild type and *coi1* similarly (Fig. 2B). These results suggest that jasmonates do not play a critical role in SO_2_-induced stomatal closure.

The effect of exogenous IAA was examined to assess the role of IAA in SO_2_-induced stomatal closure (Fig. 2C-E). Pre-opened stomata were exposed to a wide range of IAA concentrations for 2 h. The aperture width of stomata treated with 1, 10 and 100 µM IAA were similar in size (*P* > .05, Fig. 2C). Considering the dynamic as observed in the hormone quantification data, of which bidirectional time-dependent shifts in IAA levels following SO_2_ treatment were observed (Fig. 1D), we conducted a time-course analysis of stomatal aperture after the application of IAA. Time-course experiment with 10 µM IAA showed a constant stomatal aperture width over 1 h (Fig. 2D). Furthermore, we artificially create a transient IAA elevation in the first 30 min (pulse exposure) to mimic the observed changes in IAA contents after SO_2_ treatment, as in Fig. 1D. Sizes of stomatal aperture between solvent control and 10 µM IAA pulse exposure were not significantly different (Fig. 2E). We did not find a clue for the involvement of IAA in SO_2_-induced stomatal closure from this attempt. Lastly, we examined the effect of H_2_SO_3_ on the sextuple ABA receptor mutant, *sextuple*. While the mutant showed strong insensitivity to ABA application (Fig. 2F), stomatal aperture responded similar to H_2_SO_3_, as compared to the wild type (Fig. 2G). This suggests that the canonical ABA signaling mediated by the six major PYR1/PYLs/RCARs ABA receptors (Gonzalez-Guzman et al. 2012, Merilo et al. 2013) does not participate in SO_2_-induced stomatal closure. This again reject the hypothesis that ABA is involved in regulating SO_2_-induced stomatal closure, at least in *Arabidopsis*.

### SO_2_-induced stomatal closure is not mediated by hydrogen sulfide and nitric oxide in *Arabidopsis*

**Figure 3.**
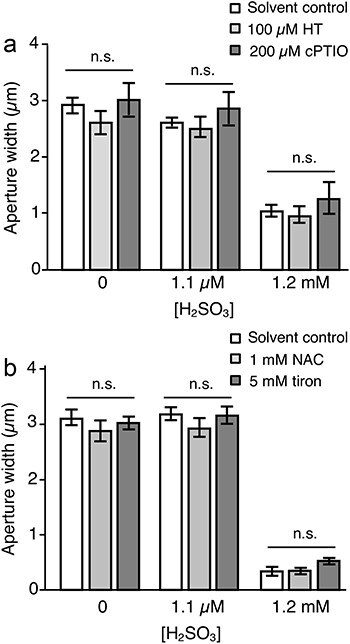
Effect of Hypotaurine (HT), 2-(4-Carboxyphenyl)-4,4,5,5-tetramethylimidazoline-1-oxyl-3-oxide sodium salt (cPTIO), *N*-acetyl-l-cysteine (NAC) and tiron (1,2-Dihydroxybenzene-3,5-disulfonic acid) on stomatal aperture width in the presence of H_2_SO_3_. Pre-opened stomata were incubated in the experimental solution containing H_2_SO_3_ added with either 100 µM HT or 200 µM cPTIO (a), and 1 mM NAC or 5 mM tiron (b), for 2 h. Four biological replicates per bar consisted of 80 stomata. All scavengers (HT, cPTIO, NAC, and Tiron) used are dissolved in purified water. ‘Solvent control’ represents H_2_SO_3_ treatments without the presence of scavengers. Error bars indicate *SE*. Results from scavenger treatments showed non-significant differences from the solvent control by Student’s *t*-test, for treatments with the same H_2_SO_3_ concentration.

It was reported that gasotransmitters such as H_2_S and NO mediate SO_2_-induced stomatal closure in *Ipomoea batatas* (Hu et al. 2014). Here, we investigated the involvement of these gasotransmitters in *Arabidopsis* using scavenging reagents, hypotaurine, and cPTIO, which abolish H_2_S and NO generation, respectively. In the absence of H_2_SO_3_, 100 µM hypotaurine and 200 µM cPTIO did not alter stomatal aperture width in *Arabidopsis* (Fig. 3A). Stomata stayed open when treated with 1.1 µM H_2_SO_3_, in all conditions, with and without the presence of scavengers. Exposure of leaves to 1.2 mM H_2_SO_3_ caused significant stomatal closure in all conditions, even in the presence of hypotaurine and cPTIO. These scavengers did not inhibit 1.2 mM H_2_SO_3_-induced stomatal closure (Fig. 3A), unlike reported in *I. batatas* (Hu et al. 2014). This suggests that SO_2_-induced signaling mechanism in guard cells which involved H_2_S and NO, is distinct between *I. batatas* and *A. thaliana*.

### Reactive oxygen species do not play a critical role in SO_2_-induced stomatal closure in *Arabidopsis*

Earlier studies have suggested that ROS formation occurs after SO_2_ exposure, and the activation of antioxidant systems is associated with resistance mechanism against SO_2_ (Asada and Kiso 1973, Rao and Dubey 1990, Madamanchi and Alscher 1991). To investigate the involvement of antioxidant systems in stomatal response of *Arabidopsis* against SO_2_, the effects of two ROS scavengers, NAC and tiron on SO_2_-induced stomatal closure were examined (Fig. 3B). Given that ROS signaling positively mediates SO_2_-induced stomatal closure, the application of ROS scavengers would inhibit stomatal closure. In the absence of H_2_SO_3_, neither NAC nor Tiron apparently affect the stomatal aperture width. In the presence of 1.1 µM and 1.2 mM H_2_SO_3_, no significant difference was observed among the scavenger treatments in any case. This indicates that ROS formation does not play a role in SO_2_-induced stomatal closure, at least in *Arabidopsis*. On the other hand, it might be one of the mechanisms resulting from SO_2_-induced injury.

### Role of CLCa proton/nitrate antiporter in guard cell resistance against SO_2_

In our previous study, we proposed that a potential mechanism of SO_2_-induced cell death in guard cells is cytosolic acidification (Ooi et al. 2019). To further elucidate this hypothesis, we conducted stomatal assays in *chloride channel a* (*clca*) mutants. CLCa transporter protein is well-known for its crucial role in facilitating proton-coupled anion transport across both plasma membrane and vacuolar membrane. However, emerging evidence suggests that CLCa also play a role in maintaining cytosolic pH homeostasis (Demes et al. 2020).

**Figure 4.**
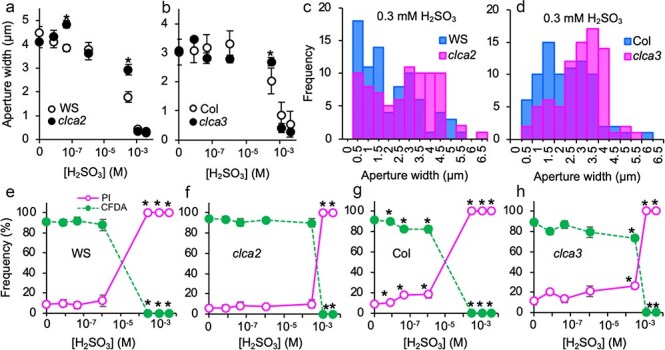
Stomatal closure and cell death induction of H_2_SO_3_ in wild-types and *clca* mutants. (a and b) Stomatal closure induction of H_2_SO_3_ in wild types (Col and WS); *clca2* and *clca3* mutants. Four biological replicates consisted of 80 stomata. Asterisks indicate significant differences (α = 0.05) by Student’s *t*-test. Error bars represent standard error of the mean. Some error bars are too small to be seen. (c and d) Distribution of stomatal aperture width in leaves treated with 3 h of 0.3 mM H_2_SO_3_. Bars represent the frequency of aperture width of wild types and *clca* mutants. Eighty stomata were measured for each line. (E–H) Guard cell viability of H_2_SO_3_-exposed wild-types and *clca* mutants. Four independent experiments consisted of 150–200 guard cells were observed for each experiment. Error bars indicate standard error of the mean. Some of the error bars are too small to be seen. Asterisks represent significant different via one-way ANOVA followed by Dunnett’s test (α = 0.05).

Using *clca* mutants (*clca2* on Wassilewskija [WS] genetic background and *clca3* on Columbia-0 [Col] genetic background), we explored the relationship between cytosolic pH homeostasis and the response of stomata to H_2_SO_3_ (Fig. 4). Stomatal aperture of wild types was narrowed by increasing concentrations of H_2_SO_3_ (Fig. 4A and B). When treated with 0.3 mM H_2_SO_3_, the aperture width became 2.04 ± 0.21 µm (WS) and 1.79 ± 0.68 µm (Col). Different from the wild types, aperture width of the *clca* mutants stayed wide at 2.69 ± 0.16 µm (*clca2*) and 2.93 ± 0.22 µm (*clca3*) (Fig. 4A and B). In the previous study, it was reported that stomatal closure of *Arabidopsis* after H_2_SO_3_ exposure consisted of two directional responses: slight widening at modest H_2_SO_3_ concentrations and tightly closed at higher H_2_SO_3_ concentrations, which bring about two separable stomatal populations (Ooi et al. 2019). That study implies that tightly closed stomatal population was of dead guard cells and the open-wide stomatal population was of viable guard cells. Here, we took a histogram analysis to investigate the stomatal populations of wild types and *clca* mutants (Fig. 4C and D). The histograms showed two phasic distributions of stomatal aperture widths. It was apparent that the frequency of wider populations was greater in the *clca* mutants than the wild types, suggesting more viable guard cells were sustained in *clca* mutants after a 3-h of 0.3 mM H_2_SO_3_ treatment. Based on our previous and current results, we infer that guard cells of *clca* mutants exhibit higher resistance to SO_2_ than the wild types. We then examined the viability of SO_2_-treated guard cells in both *clca* mutants and the wild types (Fig. 4E-H).

In accordance with our previous report, increasing concentrations of H_2_SO_3_ induced cell death of guard cells in wild types above 10^–6^ m (Fig. 4E and G, Ooi et al. 2019). In *clca* mutants, the H_2_SO_3_ concentrations that killed ∼100% of guard cells were higher compared to the corresponding wild types (Fig. 4F and H). This result is in a good agreement with the stomatal aperture width results (Fig. 4A-D), if we consider that observed stomatal closure induced by H_2_SO_3_ is associated with cell death. Furthermore, the results demonstrated in Fig. 4, collectively, suggest that sensitivity of *Arabidopsis* stomata to SO_2_ is affected by cytosolic pH homeostasis.

Given that cytosolic acidification act as the key role in stomatal closure in the exposure to H_2_SO_3_, other weak acids would also induce stomatal closure. To test that hypothesis, we assessed the effects of formic acid and maleic acid on stomatal closure in wildtypes and *clca* mutants (Supplemental Fig. S2). Maleic acid at 1.2 mM induced stomatal closure in Col and WS. This was partially attenuated in *clca* mutants. On the other hand, formic acid did not induce the closure evidently. This may be due to the difference in p*K*_a1_ of these chemicals, p*K*_a1_ of formic acid, maleic acid, and sulfurous acid are 3.75, 1.90 and 1.89, respectively. The range of p*K*_a1_ around 1.9 may efficiently acidify the guard cell cytosol, favorably kill the cells. Notably, the effect of maleic acid was attenuated in *clca* mutants (Supplemental Fig. S2), suggesting that pH homeostasis in the mutants was associated with the closure phenotype as observed for H_2_SO_3_ (Fig. 4). Unlike in the Col background, formic acid modestly induced stomatal closure in WS and *clca2*. The degree of the closure seemed comparable between WS and *clca2*. The WS background might be more sensitive to dicarboxylic acid than the Col background, and this sensitivity is not mediated via cytosolic pH. This can explain the remaining sensitivity of *clca2* mutant to 1.2 mM maleic acid. Our results suggest that weak acids with p*K*_a1_ around 1.9 can induce stomatal closure in *Arabidopsis*.

### Higher SO_2_ tolerance of *clca* stomata rendered whole-leaf phenotype to be more SO_2_-sensitive

**Figure 5.**
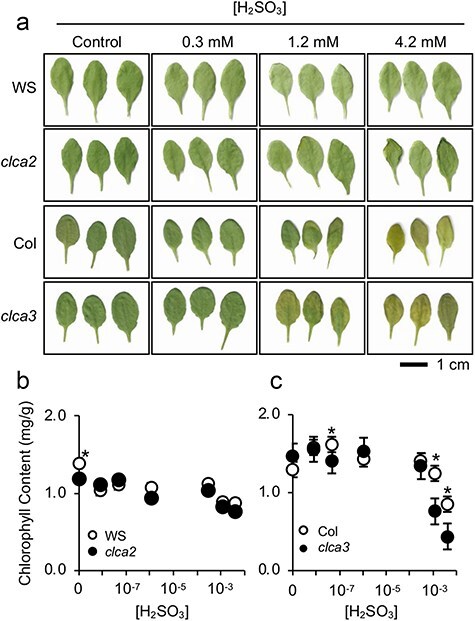
Effects of H_2_SO_3_ on the appearance of whole rosettes of wild types and *clca* mutants. (a) Representative images of excised rosette leaves after a 3 hr of H_2_SO_3_ exposure. (b) Chlorophyll content in H_2_SO_3_-treated leaves, n = 3. Asterisks indicate significant differences (α = 0.05) by Student’s t-test. Error bars represent standard error of the mean. Some error bars are too small to be seen.

Considering that stomatal closure is a stress avoidance response against hazardous gases, keeping stomatal aperture to stay widely opened can lead to a greater invasion of SO_2_ into leaf tissues, thus resulting to higher susceptibility of leaves to SO_2_. H_2_SO_3_-treated *clca* mutant leaves appeared paler than each corresponding wild type (Fig. 5A), indicating higher sensitivities of *clca* mutant leaves to H_2_SO_3_ as compared to wild types. There was no apparent difference in chlorophyll contents between WS and *clca2* (Fig. 5B). However, chlorophyll contents in *clca3* after a 3-h exposure to H_2_SO_3_ were found to be lesser than Col (Fig. 5C). There could be a minor difference in whole-leaf phenotype between wild types and *clca* mutants due to the difference in stomatal sensitivity against SO_2_. The *clca* mutants have stronger guard cell resistance against SO_2_ allowing them to stay alive and their stomata to stay open for longer time, resulting in paler leaves from larger amount of SO_2_ entrance into the leaves. Nevertheless, the discrepancy in chlorophyll content is not significant in WS and *clca2*. There may be other factors for whole-leaf SO_2_ sensitivity beside prolonged guard cell resistance gained from cytosolic pH homeostasis.

## Discussion

A potential role of ABA in mediating SO_2_-induced stomatal closure was proposed with experiments in several plant species (Kondo and Sugahara 1978, Gupta et al. 1991, Krizek et al. 2001). It is well known that not only ABA but also other plant hormones are involved in regulating stomatal closure (Murata et al. 2015). In this study, we found that contents of jasmonates and IAA in *Arabidopsis* leaves changed upon H_2_SO_3_ exposure (Fig. 1B-D). ABA contents also changed modestly (Fig. 1E). Based on these findings, we assessed stomatal phenotype in JA- and ABA-insensitive mutants (Fig. 2A, B, F and G). In addition, the response of stomata to exogenous application of IAA was also assessed (Fig. 2C-E). IAA-impaired mutants were not used since IAA-insensitive mutants show dwarfism that would hamper stomatal assay (Leyser et al. 1993, Prigge et al. 2020). The results in this study showed no sign of involvement of plant hormones in H_2_SO_3_-induced stomatal closure in *Arabidopsis*. We employed several *Arabidopsis* genotypes as study materials here using hormone-insensitive mutants available. Exploitation of mutants exhibiting hormone insensitivity in other plant species is not as easy as *Arabidopsis*. Meanwhile, *Raphanus sativus*, a Brassicaceae species was shown to be more sensitive to SO_2_ as compared to *Arachis hypogaea* (Fabaceae) and *Solanum lycopersicum* (Solanaceae) (Kondo and Sugahara 1978). Since *Arabidopsis* is a Brassicaceae species, it may exhibit SO_2_ sensitivity like *R. sativus*. Considering that there could be a potential variation in SO_2_ sensitivity among different species, a distinct phytohormone contribution to SO_2_ response may be found in other species.

It was proposed that ROS production is involved in SO_2_ toxicity (Li and Yi 2012). It is well acknowledged that ROS production plays a role in stomatal closure (Pham and Desikan 2009). Therefore, we hypothesized that ROS production following SO_2_ exposure takes part in SO_2_-induced stomatal closure. A pharmacological study showed that gasotransmitters H_2_S and NO are involved in SO_2_-induced stomatal closure in *I. batatas* and *H. fulva* (Wei et al. 2013, Hu et al. 2014). In ABA signaling in guard cells, ROS, H_2_S, and NO are postulated to coordinately function in activating Ca^2+^ channels (Honda et al. 2015). Thus, it is tempting to speculate that ROS production and successive interaction with H_2_S and NO participate in SO_2_-induced stomatal closure. In this study, evidence for the involvement of ROS in SO_2_-induced stomatal closure was not obtained based on the result from coincubation of SO_2_-treated leaves with ROS scavengers (Fig. 3B). In addition, the presence of scavengers of NO and H_2_S did not inhibit SO_2_-induced stomatal closure (Fig. 3A). Our previous study showed that SO_2_-induced stomatal closure was not impaired in the NADPH oxidase double knockout mutant, *rbohD/F* (Ooi et al. 2019). These findings suggest that the coordinate action of ROS, H_2_S, and NO does not occur in the process of SO_2_-induced stomatal closure in *Arabidopsis*. However, this does not reject the possibility that the involvement of ROS, H_2_S, and NO in SO_2_ response might be diverse across plant species.

We have anticipated that acidification of guard cell cytosol and successive cell deaths are the events contributing to the mechanism for SO_2_-induced stomatal closure. In the previous study, we showed that the toxicity of aqueous solution of SO_2_ was owing to H_2_SO_3_ but not HSO_3_^−^ or SO_3_^2−^ (Ooi et al. 2019). Given that the acidification of guard cell is critical for SO_2_-induced stomatal closure, a mutant associated with proton regulation/pH homeostasis would affect SO_2_ sensitivity of stomata. CLCa is a tonoplast-localized proton/nitrate antiporter and plays myriads of roles in plant cells. It is involved in nitrate uptake, nitrogen distribution among organic and inorganic compounds, osmotic regulation, stomatal response to abscisic acid, and pH homeostasis (Geelen et al. 2000, De Angeli et al. 2006, Wege et al. 2014, Hodin et al. 2023). In this study, we showed that the guard cells of *clca* mutants were more resistant to H_2_SO_3_ toxicity, and stomata of *clca* mutants stayed wide open with larger aperture widths as compared to the wild types upon SO_2_ exposure (Fig. 4). CLCa is shown to participate in pH homeostasis in *Arabidopsis* guard cells (Demes et al. 2020), most likely via regulating the balance of cytosolic proton and vacuolar nitrate. It is conceivable that stabilized pH homeostasis in *clca* would rescue the cell from H_2_SO_3_-induced cell death. Alternatively, it is also conceivable that enormous acidification in the cytosol by H_2_SO_3_ would result in a sudden exchange in nitrate between the vacuole and the cytosol, consequently malfunctioning cellular function via an abrupt change in ion strength and/or membrane potential in guard cells. This change in nitrate concentration may not occur in *clca* mutants since the proton/nitrate antiporter is not functional. Although the fine mechanism for the resistance of *clca* mutant guard cells to SO_2_ remained to be further clarified, our study clearly showed the SO_2_ toxicity can be mitigated by disrupting CLCa function in guard cells. Given that the biochemical role of CLCa as proton/nitrate antiporter (De Angeli et al. 2006), it is plausible that SO_2_ toxicity on guard cells and cytosolic pH are tightly related. It should be noted that some other proton-coupled transporters may also be involved in SO_2_ sensitivity through cytosolic pH homeostasis beyond CLCa. To further demonstrate that cell acidification plays a role in cell death and stomatal closure, we investigated the effects of weak acids other than sulfurous acid on stomatal aperture. Maleic acid (p*K*_a1_ = 1.9) strongly induced stomatal closure, suggesting that another weak acid capable of sufficiently acidifying the cytoplasm can trigger stomatal closure. In contrast, formic acid (p*K*_a1_ = 3.75) did not significantly promote stomatal closure. Since the p*K*_a1_ of sulfurous acid is approximately 1.9, we hypothesized that weak acids within a specific p*K*_a_ range can acidify the cytoplasm and ultimately lead to guard cell death. This model is consistent with the working hypothesis derived from experiments using the *clca* mutants. It is supported by experiments with the *clca* mutants, where the effect of maleic acid was attenuated, aligning with our working hypothesis.

Stomata serve as the gateway for SO_2_ entry into leaves, thus linking whole-leaf SO_2_ sensitivity with its stomatal phenotype. In Columbia ecotype background, *clca3* mutant demonstrated increased sensitivity to SO_2_ in terms of chlorosis (Fig. 5). This increased sensitivity may be attributed to prolonged opening of stomata at higher levels of SO_2_ due to higher resistance of guard cells of *clca3*. It is worth noting that whole leaf becomes more sensitive when guard cells exhibit greater resistance to SO_2_. Stomata close due to cell death of guard cells in response to SO_2_. Consequently, whole leaves initially appear more resistant in Col wild type in a 3-h SO_2_ exposure, although this may not be conductive to long-term survival. To enhance resistance to SO_2_ exposure, it is crucial for both mesophyll and guard cells to develop resistance to SO_2_. Additionally, it is important to recognize that the leaves of WS ecotype displayed greater resistance to SO_2_-induced chlorosis as compared to those of the Col ecotype (Fig. 5), indicating a variation in SO_2_ sensitivity even within a single species.

In this study, we observed that *clca* mutant in Col ecotype exhibits gained resistance to SO_2_ only in guard cells but not in whole leaf (Figs. 4 and 5). Nonetheless, it is conceivable that the manipulation of CLC-related genes in fruits, flowers, and mesophyll tissues could potentially lead to improved SO_2_ resistance in crops. We explored the mechanisms behind SO_2_-induced stomatal closure in *Arabidopsis*. The participation of ABA in this process was not substantiated, with observations from hormone profiling and SO_2_ treatment in a sextuple ABA receptor knockout mutant. Involvement of jasmonates and IAA were also denied via investigation employing a JA-insensitive mutant and exogenous IAA treatment (Fig. 2). Pharmacological evidence using scavengers did not support the involvement of ROS, H_2_S, and NO in the process of SO_2_-induced stomatal closure (Fig. 3). Our findings indicate the crucial role of the proton-nitrate antiporter, CLCa, in resisting SO_2_ toxicity. Reverse genetic study using *clca* mutants demonstrated lower sensitivity of guard cells against SO_2_, suggesting that acidification of guard cell cytosol is the primary mechanism leading to guard cell death and subsequent stomatal closure.

## Supplementary Material

pcaf042_Supp

## Data Availability

Sequence data used in this article can be found in the Arabidopsis Information Resource database (https://www.arabidopsis.org/) under the following accession numbers: *CLCa* (At5g40890), *COI1* (At2g39940), *PYR1* (At4g17870), *PYL1* (At5g46790), *PYL2* (At2g26040), *PYL4* (At2g38310), *PYL5* (At5g05440), and *PYL8* (At5g53160). The authors confirm that the data supporting the findings of this study are available within the article and its online supplementary materials. Additional data related to this article may be shared on reasonable request to the corresponding authors.
