# Supplementary material for: Sulfur dioxide-induced guard cell death and stomatal closure are attenuated in nitrate/proton antiporter AtCLCa mutants
Source: Plant Cell Physiol. 2025 Jun 12;66(7):1076–85. doi: 10.1093/pcp/pcaf042 (PMC12344094; doi:10.1093/pcp/pcaf042)
Supplement: pcaf042_Supp [file pcaf042_supp.zip › suppl_data/pcp-2024-e-00166-File007.pdf]

Supplemental materials

Sulfur dioxide-induced guard cell death and stomatal closure are attenuated in nitrate/proton antiporter *AtCLCa* mutants

Running head: SO_2 sensitivity and cytosolic pH

Lia Ooi[1,2]*, Takakazu Matsuura[1], and Izumi C. Mori[1,*]

[1]Institute of Plant Science and Resources, Okayama University, 2-20-1 Chuo, Kurashiki, 710-0046, Japan

[2]Plant & Microbial Research Unit, Research, Technology & Value Creation Division, Nagase Viita Co., Ltd., 675-1 Fujisaki, Naka-ku, Okayama 702-8006, Japan

*Corresponding authors

Izumi C. Mori, imori@okayama-u.ac.jp

Lia Ooi, ooi.lia@nagase.co.jp

This file contains:

2 Supplemental Figures

1 Supplemental Table

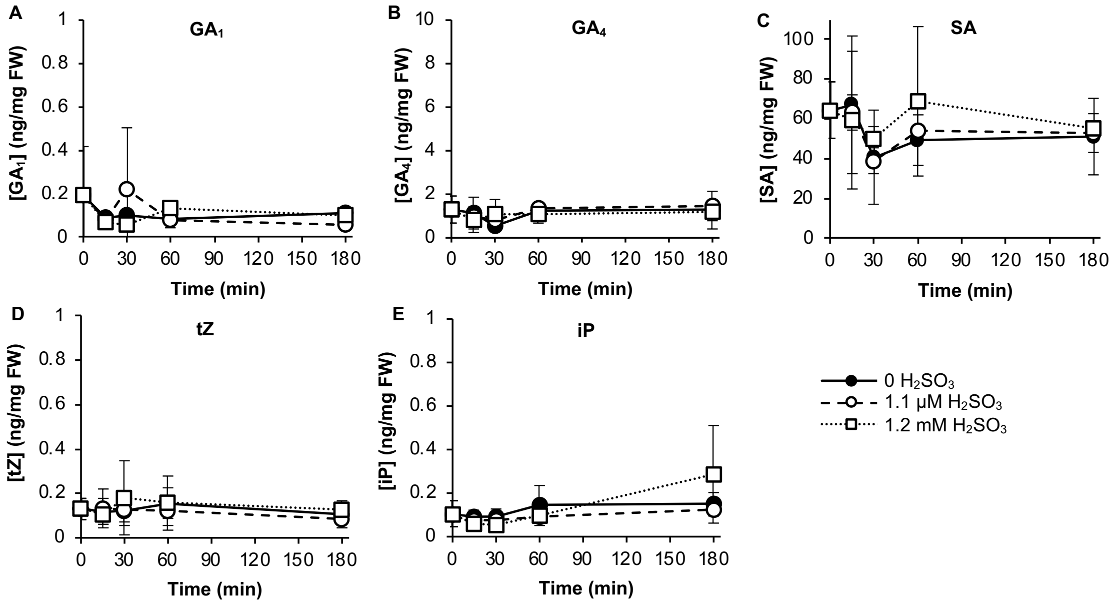

Supplemental Figure S1. Contents of gibberellin A_1 (GA_1) and gibberellin A_4 (GA_4), salicylic acid (SA), zeatin (tZ) and isopentenyladenine (iP) in H_2SO_3-treated leaves. Mature rosette leaves of wild-type plants were incubated in stomata opening buffer containing 0 (control), 1.1 μM, and 1.2 mM l^{-1} H_2SO_3 for 180 min under white light radiation (n=6). Error bars represent *SD*, some error bars are too small to be seen. One-way ANOVA followed by Tukey's honestly significant post hoc test was conducted for each data identified no significant difference (α = 0.05) for each dataset.

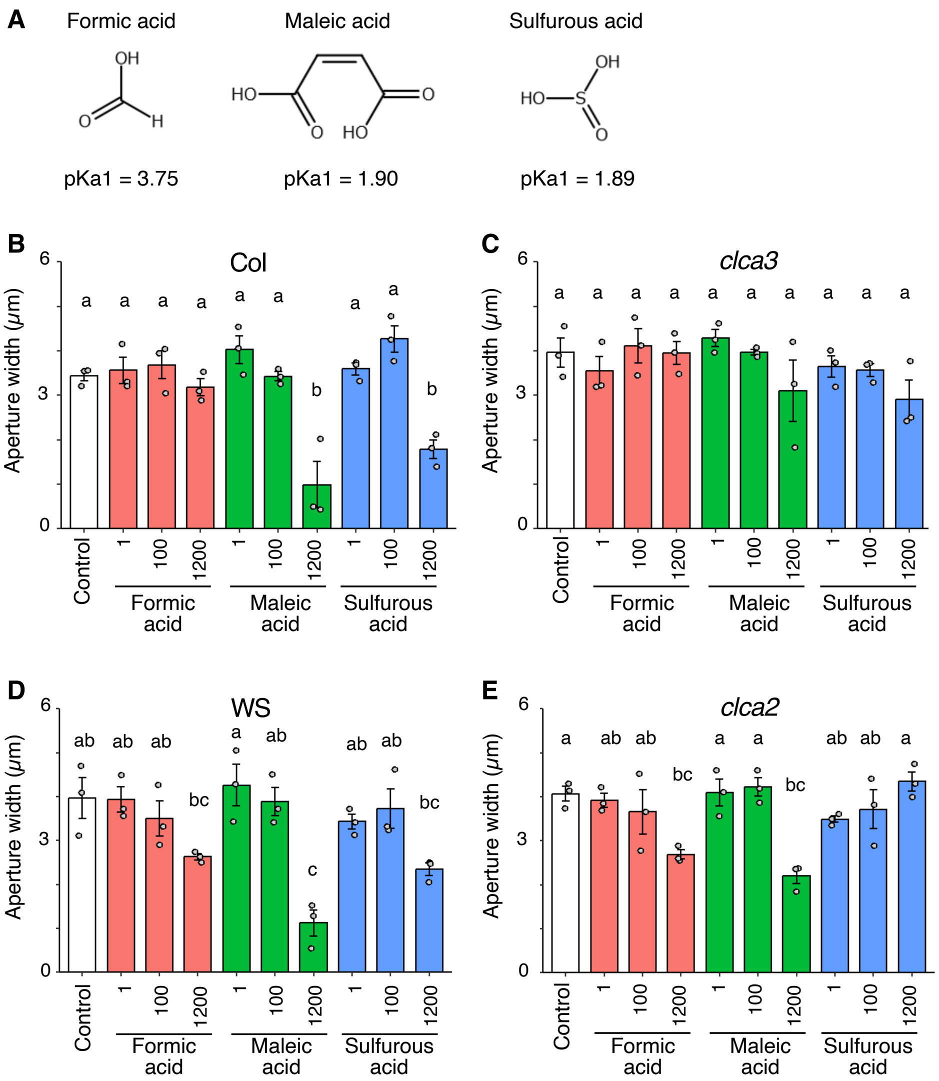

Supplemental Figure S2. Effects of formic acid and maleic acid on stomatal aperture. (A) Structure and pK_{a1} of formic acid, maleic acid and sulfurous acid. (B to E) Concentrations of chemicals are shown as µM under the X-axis. Error bars represent *SE* (n =3). Small letters indicate significance tested by Tukey-Honestly Significant Difference test (n = 3). Control indicates treatment with water.

Supplemental Table S1 Concentrations of the protonated chemical species and pH of formic acid and maleic acid solutions prepared in the opening solution made up of 10 mM MES-Tris buffer solution.

	C_{total} (mM)	pH (mean ± standard deviation, n = 3)	$C_{protonated}$ (μM)
Formic acid	0.1	5.70 ± 0.00	1.1
	1.7	4.90 ± 0.02,	110
	3	3.92 ± 0.10	1200
Maleic acid	2.1	5.10 ± 0.00	1.1
	3.0	3.18 ± 0.01	150
	7.3	2.57 ± 0.05	1200

C_{total}, nominal concentration of total formic acid and maleic acid added to the buffered solution. $C_{protonated}$, calculated concentration of the protonated form with the measured pH of the solution and pK_{a1}. Values of pK_{a1}: 3.74, formic acid; 1.90, maleic acid.
